# Marker-free image registration of electron tomography tilt-series

**DOI:** 10.1186/1471-2105-10-124

**Published:** 2009-04-27

**Authors:** Carlos Oscar Sanchez Sorzano, Cédric Messaoudi, Matthias Eibauer, JR Bilbao-Castro, R Hegerl, S Nickell, S Marco, JM Carazo

**Affiliations:** 1 Escuela Politécnica Superior, Universidad San Pablo-CEU, Campus Urb Montepríncipe s/n, E-28668 Boadilla del Monte, Madrid, Spain; 2 Biocomputing Unit of the National Center of Biotechnology (CSIC), Madrid, Spain; 3 Institut Curie, Centre de Recherche, Orsay, F-91405, France; 4 INSERM, U759, Orsay, F-91405, France; 5 Max-Planck Institute for Biochemistry, Martinsried, Germany; 6 Computer architecture department, Univ de Almería, Spain

## Abstract

**Background:**

Tilt series are commonly used in electron tomography as a means of collecting three-dimensional information from two-dimensional projections. A common problem encountered is the projection alignment prior to 3D reconstruction. Current alignment techniques usually employ gold particles or image derived markers to correctly align the images. When these markers are not present, correlation between adjacent views is used to align them. However, sequential pairwise correlation is prone to bias and the resulting alignment is not always optimal.

**Results:**

In this paper we introduce an algorithm to find regions of the tilt series which can be tracked within a subseries of the tilt series. These regions act as landmarks allowing the determination of the alignment parameters. We show our results with synthetic data as well as experimental cryo electron tomography.

**Conclusion:**

Our algorithm is able to correctly align a single-tilt tomographic series without the help of fiducial markers thanks to the detection of thousands of small image patches that can be tracked over a short number of images in the series.

## Background

Electron tomography is a rapidly growing technique that produces structural information of organelles and cell compartments at a resolution between 40 and 20Å [1-3]. This kind of information helps structural biologists to understand how macromolecular machines interoperate in the cell to perform their functions and is giving rise to what is called "visual proteomics" [4,5].

This technique relies on the tomographic reconstruction of the object under study from the projection images acquired by the electron microscope. The most popular data collection geometry is a single-axis tilt series [6], although other geometries are also possible like multiple-axis [7-9] or conical tilt [10,11]. In this article we concentrate on single-axis tilt series. With this technique the sample is tilted around an axis to provide different projection views of the object under study. As the object is tilted electrons have to traverse thicker sections. Thus, this technique is normally restricted to a maximum tilt of about 70°. Due to mechanical instabilities of the sample holder during rotation, it is possible that some of the images of the series exhibit a large shift with respect to the rest. Besides their shift, their relative projection directions must be established before entering the three-dimensional reconstruction process. This basically amounts to determining the tilt axis in each of the images since its exact position may not be the same on each projection.

Alignment of the tilt series is usually performed by aligning gold beads that serve as fiducial markers [12-14]. Although quite effective when these markers are available, this technique is not always applicable since markers are not always visible or trackable, or because, simply, they are not available [15]. Alternatively, image corners can be used as fiducial markers [16], or the image information content itself can be used through cross-correlation [17,18]. The image information has been proven to be the most valuable information in image registration [19] and, therefore, algorithms based on cross-correlation with a reference volume or similar techniques are supposed to provide the best results as is the standard case in electron microscopy of single particles [20]. However, when aligning tilt series there is no *a priori *volume that can be used as reference, and for this reason, the algorithm employed must align the images with information exclusively contained in the image series. There are three main possibilities to use cross-correlation in this context of tilt series alignment. The first possibility is to serially align the first image with the second by cross-correlation, the second with the third, etc. This approach compares similar images, although it has the drawback of serious potential drifts (by error propagation) of the alignment parameters resulting in inaccurate estimations of the tilt axis and shift parameters in each of the images. The second option is to use cross-correlation to track features within the tilt series. Features are defined as fiducial points in the images (due to change of contrast, borders, corners, etc.) that are searched by cross-correlation in neighboring images. The feature tracking allows to define markers that are visible in only a subsequence of the whole tilt series. The third approach constructs a rough reconstruction of the tomogram and realigns the tilt series with respect to reprojections of the rough reconstruction [18].

We exploit the second option by searching for image regions that can be tracked within a subseries of the tilt series. An overall affine image registration is performed to be able to predict the position of any image region in any of the adjacent images. If all images are numbered according to their tilt angle in the tilt series, a region of image *i *is sought in image *i *+ 1, first predicting its position through the affine transformation, and then by local refinement of the cross-correlation. Once, the region is found in image *i *+ 1, the equivalent region in image *i *+ 1 is sought in image *i *+ 2. The algorithm thus propagates a coordinate in image *i *to images *i *+ 1, *i *+ 2, until the region sought cannot be found anymore because it becomes obscured by other objects, it changes its shape, or the noise does not allow to find it. The same is done backwards. All the coordinates corresponding to equivalent image regions form a landmark chain which most likely does not cover the whole tilt series. Landmark chains are further refined to guarantee that they correspond to equivalent image regions. Finally, all landmark chains are used to perform a robust regression of the image alignment parameters. An advantage of this approach is that it is fully automatic and it can produce thousands of landmark chains which are used to align the images. We have tested our algorithm with synthetic data as well as experimental cryo electron microscopy data.

The method proposed in this paper differs from other published methods in a number of ways. The algorithm proposed by Penczek *et al*. in [9] is very similar in its basics to the one presented in this paper. Our main differences are that we provide a mechanism to automatically estimate landmark positions, our algorithm is based on the use of thousands of short landmark chains (although this feature is also allowed in Penczek's algorithm, it was not conceived to be used in this way), we perform a robust estimation of the alignment parameters, and we provide matrix closed forms of the parameter estimates which are more convenient for implementation. The algorithm described by Mastronarde in [21] differs also mainly in our automatic estimation of the landmark positions yielding thousands of short landmark chains. The optimization procedure is also different, ours being more analytical. The approach of Brandt in [22] and Castaño-Díez in [23] differ from ours in the way landmark chains are automatically detected and refined, and the posterior optimization procedure followed to estimate the alignment parameters. Their algorithms also perform a robust optimization, although in a different way to ours.

## Results and discussion

Before reading this section we strongly suggest to read the Methods Section to get acquainted with the terminology and different parameters of the algorithm.

### Simulated data

**Figure 1:**
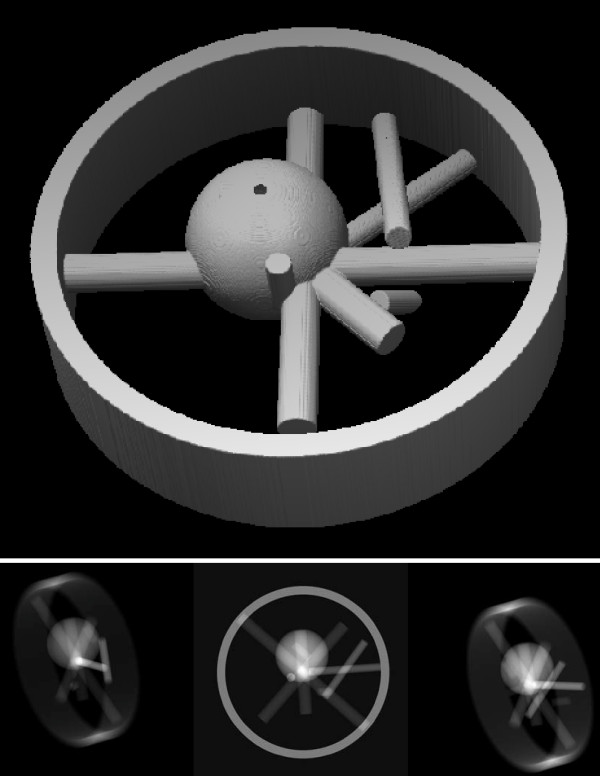
**Phantom data**. Top:Isosurface of the phantom used for the simulated data. Bottom: Projections of this phantom at -60°, 0°, and 60°. The tilt axis forms 78° with the horizontal axis. These images correspond to experiment number four where a shift drift was simulated along the tilt series. This explains the different location of each of the projections.

In order to test the accuracy of the new alignment algorithm we created a phantom built with mathematical descriptions of cylinders and spheres (see Fig. 1). The phantom size is 512 × 512 × 128 voxels. For the first experiment 41 images of size 512 × 512 were simulated with a maximum tilt of 60° and a step of 3° between images. The tilt axis passed through the coordinate origin and its direction was defined by the angles *α *= 0 and *β *= 90 (see Methods). No in-plane transformations were applied to the projections. Landmark regions had a size of 7.5% the total size of the images (regions were 39 × 39 pixels in this case), a grid of 40 × 40 evenly spaced points in each projection image were considered as centers of the landmark regions (this means that between two adjacent regions of length 39, there is an overlap of 27 pixels).

Landmark chains were accepted if the correlation coefficient between any two regions being compared was higher than 0.98, and the total length of the chain was larger than 10 images. 5801 landmark chains were identified with an average length of 16.5 images. 3136 landmark chains were used in the last regression iteration. There was an average error between the projection of the 3D landmarks and its observed projections of 0.86 pixels (i.e., the goal function in Eq. 6 divided by the total number of 3D landmarks). To objectively evaluate the quality of the reconstruction performed with the alignment parameters produced by our algorithm, we computed the correlation index of this volume with the volume reconstructed with the ground-truth alignment parameters. The result was 0.98.

For the second experiment 61 images were simulated with a maximum tilt of 60° and a step of 2° between images. The rest of the conditions were as in the previous example except that *α *= 78 (this experiment was done in order to test the algorithm ability to find a tilt axis that is not aligned with any of the standard axes). The parameters for our algorithm were identical as in the first experiment. 16562 landmark chains were identified with an average length of 23.1 images. 8748 landmark chains were used in the last regression iteration. There was an average error between the projection of the 3D landmarks and its observed projections of 0.51 pixels. The correlation between the volume reconstructed with the estimated parameters and the reconstruction performed with the ground-truth parameters was 0.99.

In the third experiment, images were collected as in experiment 2 except that *α *= 0. The collected images were randomly shifted in X and Y by a displacement following a Gaussian distribution with zero mean and standard deviation 20 (note that the images were of size 512 × 512). 12048 landmark chains were identified with an average length of 21.38 images. 6284 landmark chains were used in the last regression iteration. There was an average error between the projection of the 3D landmarks and its observed projections of 1.13 pixels. The correlation between the volume reconstructed with the estimated parameters and the reconstruction performed with the ground-truth parameters was 0.98.

The fourth experiment was aimed at characterizing the performance of the algorithm under slowly varying drifts. The 61 images of the second experiment were used for this last experiment. The first image was rotated -4 degrees, the second image was rotated -2 degrees, the third image was not rotated, the fourth image was rotated 2 degrees, and the fifth image was rotated 4 degrees. This sequence (-4, -2, 0, 2, 4) was repeated along the rest of the series. After rotation, the first image was shifted -30 pixels in each direction. Then, the rest of the images were shifted one pixel vertically and horizontally with respect to its predecessor so that the last image was shifted 61 pixels with respect to the first one. Our algorithm was run with chain lengths of 5, a correlation threshold of 0.99, local regions of a size of 7.5% the total size of the images. 28843 landmark chains were identified with an average length of 17.74 images. 14814 landmark chains were used in the last regression iteration. There was an average error between the projection of the 3D landmarks and its observed projections of 0.33 pixels. The correlation between the volume reconstructed with the estimated parameters and the reconstruction performed with the ground-truth parameters was 0.99.

In the last experiment, we checked whether the algorithm was able to work in a noisy environment. The experimental setup was the same as in the previous experiment but we added white noise to the simulated images (the level of noise added was such that the correlation between pairs of consecutive images in the tilt series was in the same order of the correlation observed between consecutive images in the experimental tilt series described below for the *Pyrodictium abyssi*). The presence of noise significantly lowers the correlation between patches. For this reason we reduced the patch size to only 4% of the image size and lowered our correlation threshold to 0.9. 7848 chains were automatically extracted, out of which 4134 participated in the last regression iteration. The average of the error between the projection of the 3D landmarks and the observed projections was 2.7 pixels. Finally, the correlation between the volume reconstructed with the estimated alignment parameters and the reconstruction performed with the ground-truth parameters was 0.94.

### Experimental data

**Figure 2:**
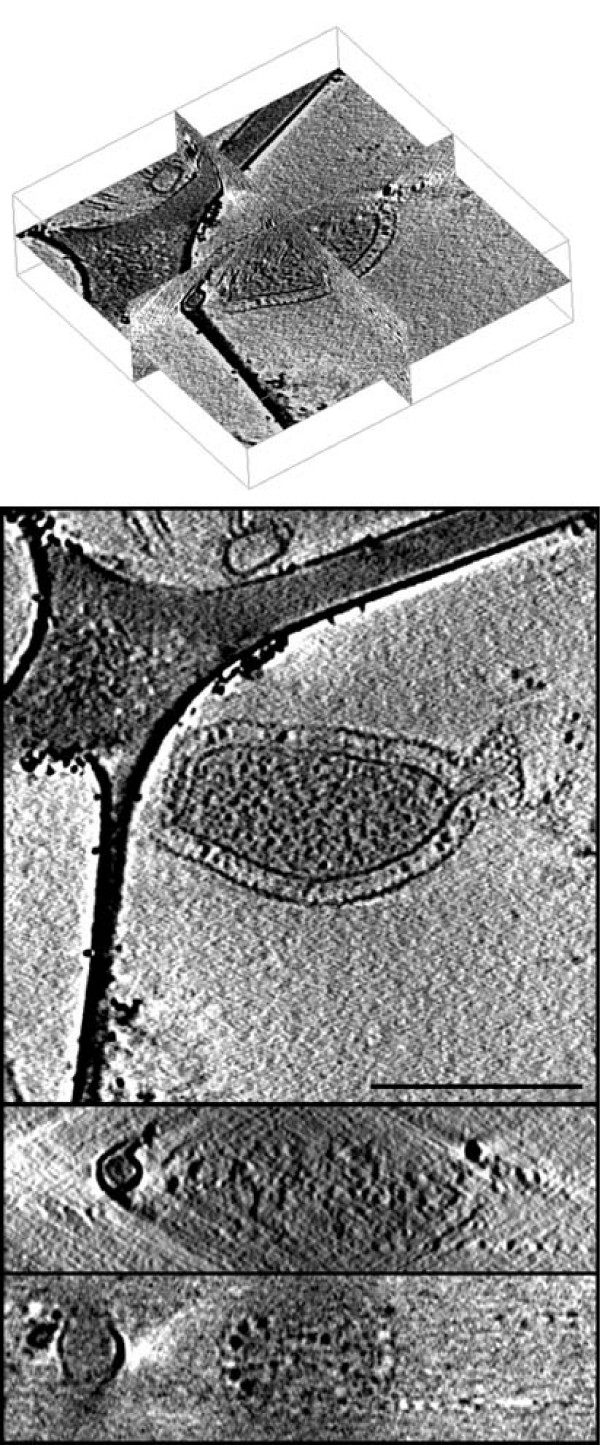
**Experimental data reconstructed automatically**. Three orthogonal slices of the *Pyrodictium abyssi*. The three slices are shown in 3D with their relative orientations (top) as well as separately. The scalebar represents 500 nm.

**Figure 3:**
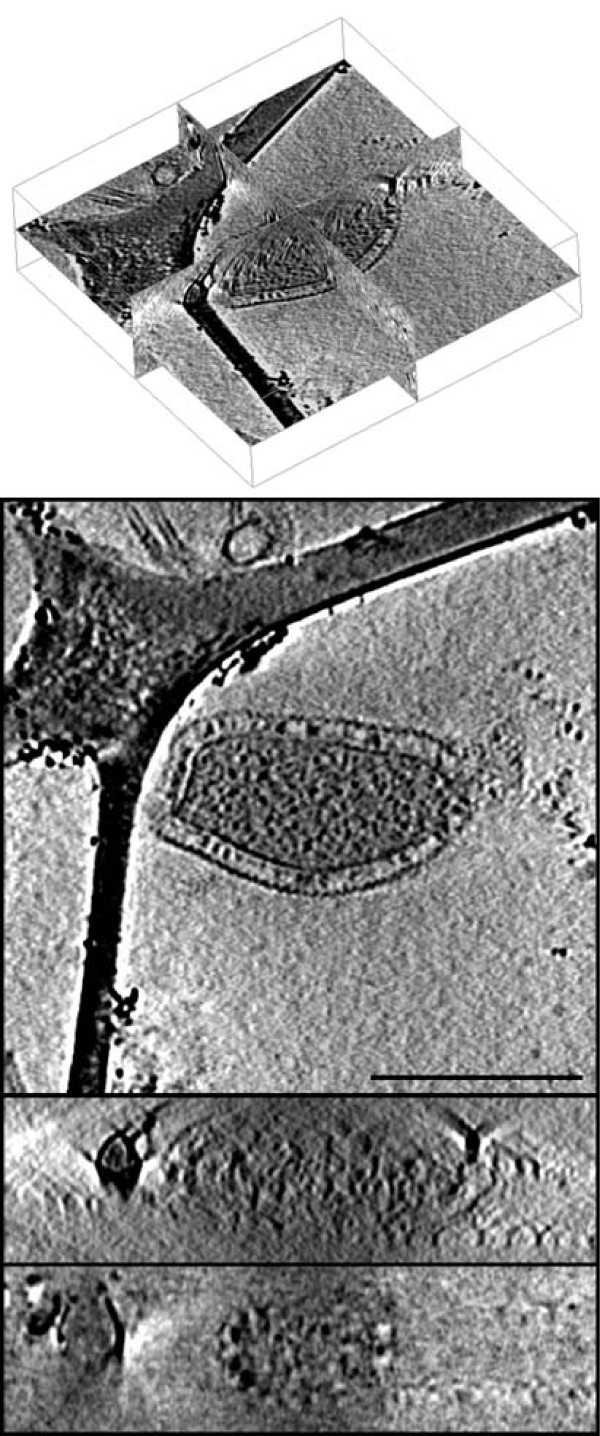
**Experimental data reconstructed automatically**. Top: Corresponding orthogonal slices of the volume in Fig. 2 which was reconstructed using the parameters estimated by the proposed automatic alignment algorithm. Bottom: Reconstruction of the same dataset used in Fig. 2 aligned by manually selecting the fiducial markers instead of using the automatic alignment algorithm presented in this paper.

**Figure 4:**
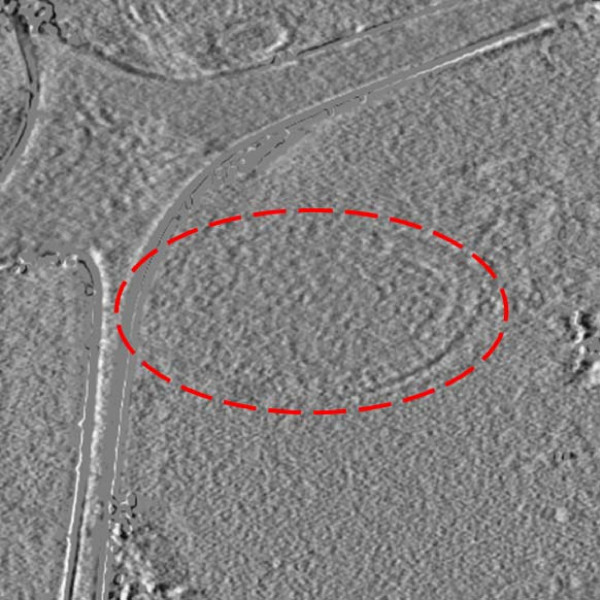
**Slice of the difference between the two volumes shown in Fig. 3**. As can be seen most of the differences between the two volumes are not located where the bacteria is.

For testing the algorithm with real data a tilt series of a *Pyrodictium abyssi *cell strain TAG11 [24], which was embedded in a vitreous ice layer [25] on a holey carbon-coated grid, was used. Electron tomography was performed using a CM120 Biofilter (FEI, Eindhoven, The Netherlands) at an accelerating voltage of 120 keV. It was equipped with a postcolumn energy filter (Gatan, Pleasanton, CA), operated in the zero-loss mode [26], resulting in increased image contrast [27]. The tilt series ranged from -70° to +67° with a tilt increment of 1.5° and a total number of 91 images. Data acquisition was carried out using fully automated procedures under low-dose conditions [28]. The images, which were 1024 × 1024 pixels each, were recorded at 14,500× magnification, with a pixel size at the specimen level of 1.62 nm. The defocus was set to -10 *μ*m; the first zero of the phase-contrast transfer function was at (5.8 nm)^-1^. The images collected were not lowpass filtered or deconvolved with the microscope contrast transfer function. Our algorithm was run with chain lengths of 5, a correlation threshold of 0.9, local regions of a size of 4% the total size of the images (patches of size 41 × 41 pixels, with an overlap of 15 pixels). 1255 landmark chains were identified with an average length of 15.65 images. 706 landmark chains were used in the last regression iteration. There was an average error between the projection of the 3D landmarks and its observed projections of 0.85 pixels. The resulting reconstruction can be seen in Fig. 2. For comparison purposes we performed a reconstruction from the same dataset using the standard alignment algorithm based on manual selection of fiducial markers which is shown in Fig. 3. For the manual alignment four landmarks were identified along the whole tilt series and TOM toolbox was used for the alignment and 3D reconstruction [29]. As can be seen, the two reconstructions are not significantly different, although they are not identical. The correlation between the two volumes after registration was 0.635, however, as can be seen in Fig. 4, these differences are not located where the archaeobacteria is and are mainly located in the surrounding structure (this is most likely due to the fact that the manually selected landmarks were chosen from the bacteria region while the surrounding objects were disregarded).

## Discussion

In this article we have presented a new algorithm for the automatic alignment of electron tomographs. The main idea is the identification of thousands of small landmark chains that clearly correspond to the same point in a number of images. In contrast to previously published papers, our objective is to detect many short chains instead of a few long ones. This has the advantage of providing much more information to the alignment process and simplifies at the same time the task of identifying corresponding points within the tilt series. In fact, that is currently one of the limiting steps in image alignment in electron tomography: even if gold beads are available (which also result in stripe artifacts present in the 3D reconstruction that may hide interesting structural features), sometimes they are so much clustered that it is difficult to identify the same gold bead within the whole tilt series since in some of the images it is among many other gold beads clustering in the same region. Moreover, this process of identifying corresponding landmarks within the tilt series is many times done by the user, or done automatically by cross correlation of correlative image pairs and refined/corrected by the user. The main reason for the need of user interaction is that tracking the same point within the whole series is a difficult problem to be solved automatically. Placing the focus on thousands of short landmark chains makes the problem much more computationally tractable and reliable. Furthermore, the robust regression step tends to remove those possibly wrongly identified chains.

Our treatment of the chain identification is quite strict and it is one of the keys of the algorithm. The idea is to have a first rough idea of corresponding landmarks using the affine transformation between any pair of correlative images. Given the size of the images there is enough information to determine the six parameters of the affine transformation quite faithfully. Moreover, a careful global and accurate search of the parameters is performed through the combined use of a global optimizer (differential evolution) and a local optimizer (Powell's conjugate gradient method). This initial guess of the landmark chain is improved by locally shifting the image regions to maximize the correlation between corresponding regions. This allows for elastic local deformations that cannot be taken into account solely by the affine transformation (note that the elastic deformations are considered at the chain construction level, although the whole alignment procedure can only explain affine transformations and, therefore, it looses part of the richness of information captured by the landmark chains). Once the landmark chain is identified by using correlative image pairs, it is carefully refined by forcing each region to be similar not only to the equivalent regions in adjacent images, but to equivalent regions all along the image chain. This is also one of the key steps of the algorithm that greatly improve the landmark chain identification, and discards many slowly drifting chains. For the success of the algorithm it is recommended that a "dense" grid of landmark chains is available, meaning by this that there are enough landmark seeds in the image (we chose in our experiments a grid of 40 × 40) so that the local significantly overlap (in our experiments we used patches that overlapped between 35% and 70% of the patch length). We also suggest to use as small local patches as possible (values between 3% and 5% of the image size are sensible). Poor coverage of the image by the local patches will most likely result in the miss of many landmark chains, and consequently missing much of the information available in the image series.

Finally, our derivation of the equations governing the optimization of the alignment parameters, although equivalent to the previous derivation of Penczek *et al*. [9], is much more compact and provides further insight in the nature of the multivariate optimization. This fact facilitates the algorithmic implementation of the optimization procedure.

## Conclusion

In this paper a new algorithm for automatic alignment of electron tomographs has been presented. The algorithm is based on the automatic detection of thousands of short landmark chains identifying image regions that clearly correspond to the same point in a relatively small number of images. Regions identified in this way must correlate well (usually with a correlation coefficient higher than 0.9) among all the corresponding pieces in the landmark chain, and the average of all of them. After these corresponding regions have been identified, a robust regression process is performed to finally find the relative alignment between all images. The process has been shown to successfully align images under conditions of random shifts between image pairs, and under conditions of slow but sustained drift between images. It has also been successfully applied to experimental images. The algorithm is available from the Xmipp package [30] and will be soon incorporated into TomoJ [31].

## Methods

### Affine registration of two images

Given any two images *I*^(*i*) ^and *I*^(*j*) ^the affine registration tries to find an affine matrix

(1)

transforming homogeneous coordinates (**t**_*H*_) of the image *i *into the image *j*. We look for this affine transformation by maximizing the cross-correlation of the images registered bidirectionally [32,33]

(2)

The cross-correlation in each direction is carefully computed only in those regions which are visible in both images, i.e., only at those coordinates **t**_*H *_such that **t**_*H *_∈ Ω_*i *_and *A*_*ij*_**t**_*H *_∈ Ω_*j*_, being Ω_*i *_and Ω_*j *_the set of coordinates where the images *i *and *j *are defined.

The maximization problem in Eq. 2 is solved using two different optimizers: Differential Evolution (DE) [34] and Powell's conjugate gradient method [35]. DE is a global optimizer based on genetic algorithms. It is rather good at approximately finding the best affine transformation between any two images. However, its convergence once it is closed to the global optimum is rather slow. Then we change to a fast local optimizer such as Powell's conjugate gradient method. In this way, we have the advantages of a global optimizer with a fast, locally convergent algorithm.

### Landmark chains

In our algorithm, image regions act as landmarks. Therefore, we must track image regions along the tilt series. However, since it is rather unlikely that the same region can be tracked along the whole series, we allow for landmark chains that start at a given image of the tilt series and finish in some other image. The region being matched along the chain need not to be detectable in all images in between the first and last image.

Every image in the tilt series, *I*^(*i*)^, is divided in a fine grid of overlapping image regions of a size much smaller than the whole projection. The center of each region is a landmark candidate. Given a region in image *i*, this region is sought in image *i *+ 1, first by predicting its position using the affine transformation *A*_*i*,*i*+1 _computed in the previous section, and then by locally optimizing the correlation index between the two corresponding regions. If the correlation index is greater than a given threshold, then the region in image *i *+ 1 is accepted as the one matching the landmark region in image *i*. The new region in image *i *+ 1 is sought in image *i *+ 2, and the process is subsequently repeated until the correlation threshold criterium is not met. The same procedure is performed backwards. At the end of this process we have a landmark chain extending from image *i *- *N *to image *i *+ *M *for some integers *N *and *M*. If the landmark chain is shorter than a certain user-supplied threshold, the chain is discarded.

If the landmark chain survives the previous filter, it is refined in an iterative way in order to avoid coordinate drifts due to the sequential search of regions. For all images between *i *- *N *+ 2 and *i *+ *M *the coordinate of the landmark in image *l *is refined with respect to that in image *l *- 2. This correction runs forward in the chain. When it finishes, the positions are corrected backwards (the position at image *l *is refined with respect to that of image *l *+ 1) running from *i *+ *M *to *i *- *N *+ 1. This process is repeated with forward steps of 3, 4, ... up to a value selected by the user (i.e., the landmark at image *l *is refined with respect to the image *l *- 3, *l *- 4, ... respectively), while the backward correction is always performed with a step of 1. After this correction loops, the average of all regions in the chain is computed. Those regions that do not meet the correlation criterium with the average region are removed from the chain. Then the whole refinement step is repeated once again with the surviving images. Those chains whose length after refinement is smaller than the specified threshold are discarded.

### Optimization of the 3D landmarks and the image in-plane rotation and shift

Once a list of landmark chains is produced in the previous section, each chain is assumed to correspond to the projection of the same 3D region, whose center is projected onto the center of each region. The 3D coordinates of these 3D landmarks as well as the rigid transformation parameters explaining the rotations and shifts of each projection in the tilt series are computed by robust regression. Let **r**_*j *_be the 3D coordinate of the *j*-th landmark and let **p**_*ij *_be the 2D coordinate of its projection onto image *i*. Let *V*_*j *_be the set of all images on which the *j*-th landmark is seen (remind that the landmark chain may not contain all images between *i *- *N *and *i *+ *M*). Then, the relationship between **r**_*j *_and **p**_*ij *_can be expressed as

(3)

where *A*_*i *_is a projection matrix accounting for the tilting around the tilt axis (which may have any arbitrary orientation in the plane perpendicular to the electron beam) and a posterior in-plane rotation, and **d**_*i *_is a 2D vector accounting for an in-plane shift of image *i*. *A*_*i *_is computed as

(4)

where *H *is a matrix projecting a 3D coordinate onto the XY plane,  is a rotation matrix of *ψ*_*i *_degrees around the *Z *axis (note that the *Z *axis is the beam axis, therefore, this represents the in-plane rotation particular to each image), and  is a rotation matrix of *θ*_*i *_degrees (the tilt angle of each image) around the tilt axis **u**_*axis*_. The tilt axis is described by two Euler angles *α *and *β *corresponding to a rotation around the *Z *axis and, then, a rotation around the new *Y *axis. The vector representation of the direction of the tilt axis is

(5)

Note that this projection model assumes that the tilt axis passes through the coordinate system origin. In practice this is seldom the case, although as shown later in the Appendix, the tilt axis can be arbitrarily placed in the tilt series as long as the aligned projections are consistent with the assigned position. The regression problem is to minimize the error between the projections of the 3D landmarks experimentally observed and their projections under theoretical tilting and in-plane rotation and shift

(6)

or what is the same

(7)

where *V*_*i *_is the set of landmarks detectable in the image *i*.

This regression problem is linear in **d**_*i *_and **r**_*j *_and non-linear in *ψ*_*i*_, *α *and *β*. To find the minimizer of Eq. 7 we decompose the problem in two nested searches. The first search explores the *α*, *β*-space. For each combination of *α *and *β *proposed in a first stage by an exhaustive grid search and in a second stage by Powell's conjugate gradient method whose initial solution is the minimizer of the exhaustive grid search, the minimum is sought in the *ψ*_*i*_, **d**_*i *_and **r**_*j *_parameters. The minimum in these three parameters are sought in an iterative way by assuming all the rest of the parameters fixed and minimizing the goal function (*E*) with respect to the parameter treated at each time. Note that this optimization procedure may be trapped in a local minimum and that this optimization process is different from the one performed to find the affine transformation previously described. It can be easily shown (see next section) that

(8)

In the same way,

(9)

where  is the average of all landmark projections in image *i *and  is the average of all the current estimates of the 3D landmarks visible in image *i*. Finally (see next section),

(10)

and

(11)

### Derivation of the matrix form of the regression problem

In this section we derive in a compact form the minimization of the regression problem. Let *E *be the goal function in Eqs. 6 and 7

(12)

We compute the derivatives of this goal function using the vector derivatives  and . From these rules, it is easy to derive

(13)

being *N*_*i *_the number of elements of *V*_*i*_, i.e., the number of 3D landmarks visible in image *i*. From this last equation it follows

(14)

Now deriving with respect to **r**_*j*_

(15)

from which

(16)

Considering that , and given the specific nature of *H *and , *A*_*i *_can also be expressed as . We will refer to  as . Then, the goal function *E *can be expressed as

(17)

Now we compute the derivative of *E *with respect to  using the matrix derivative rule 

(18)

Taking into account that a clockwise rotation matrix is given by , then

(19)

From which it can be easily derived

(20)

### Robust optimization

The optimization procedure described in the previous sections is firstly run with all landmark chains detected. Then, the fitting error is computed for each landmark chain *j *as

(21)

A user-supplied percentage of the chains with worse fitting errors are removed from the list of landmark chains assuming that they may correspond to chains that have been wrongly computed, and all the parameters (direction of the tilt axis, position of the 3D landmarks and image in-plane rotation and shifts) are reestimated with the chains remaining. This process is repeated three times, in this way a robust fitting of the alignment parameters is performed.

## Authors' contributions

COSS designed, implemented and tested the algorithm. CM participated in the design of the algorithm and is porting it from Xmipp to TomoJ. JRB helped with the parallelization of the algorithm. ME and SN provided the experimental data for the tests and interpreted the results. RH provided useful discussions in the early stages of the algorithm design. SM and JMC revised the manuscript and provided useful suggestions during the algorithm development. All authors read and approved the final manuscript.

## Appendix

### On the exact location of the tilt axis

The projection model used in this article is

(22)

which assumes that the tilt axis passes through the coordinate system origin. However, in practice this is seldom the case, and one may wonder if it would be possible to estimate its location with respect to the microscope setup. In this appendix we show that this is not possible. This ambiguity, more than a drawback, is an advantage since it allows us centering the tilt series at will. Finally, we propose a sensible centering of the tilt axis so that the image at zero degrees (or the one closest to this tilt angle) is unmoved. However, the reader is free to locate the tilt axis to his liking.

Let us consider the full projective model

(23)

where **r**_0 _is a point of the tilt axis in 3D space and, therefore, the tilt axis is not forced in this way to passthrough the coordinate system origin. Let us add a constant value **r**_*bias *_to the tilt axis

(24)

This means that any bias we add to **r**_0 _can be automatically compensated by the appropriate shift  yielding the same regression residuals. This means that absolutely locating the tilt axis within the microscope is hopeless.

From another perspective this also permits us to arbitrarily locate the tilt axis in the region of interest as is usually done, to the best of our knowledge, with no formal proof. In particular, we here propose a methodology to locate the tilt axis such that the image at zero degrees of tilt (or the one closest to this value) does not move. This can be easily done by setting **d**_*i *_= **0 **for the *i *corresponding to the unmoved image. In the following, we will refer to this image as *i *= 0 even if its tilt angle is not exactly zero but a small number *θ*_0_. The rest of the **d**_*i*_'s in the optimization process will be accordingly adjusted to achieve the minimum regression residual. However, setting **d**_*i *_= **0 **only consumes two of the three degrees of freedom provided by **r**_*bias*_. We propose to determine the extra degree of freedom in the following way. Suppose that  (i.e., the tilt axis is perpendicular to the electron beam as is usually the case). Then, the images in the tilt series can be aligned so that the tilt axis is parallel to the Y axis (the vertical axis in the projection images) by applying

(25)

The extra degree of freedom is manifested by a tilt axis that may not be within the volume to be reconstructed (i.e., the tilt axis is well above or below the volume to reconstruct). This can be easily detected because the corrected 3D landmarks show a positive or negative mean Z component. The projection of the 3D landmarks seen at image 0 in the aligned tilt series is given by

(26)

These are the projections of the original 3D landmarks, rotating around the original tilt axis and subsequently corrected. However, we can find new 3D landmarks, rotating around the image vertical axis (after alignment of the images this is the new tilt axis) whose projections are the same as the ones found at image 0. It can be easily proved that the new 3D landmarks can be defined as

(27)

under the projective model

(28)

in which there is no in-plane shift or rotation since these effects have already been corrected. Under these conditions, we propose to locate the tilt axis in a position such that **r**_0 _= (0, 0, *z*_0_)^*t*^. Then, the previous projective model has to be changed to

(29)

In order to make the tilt axis pass through the middle of the reconstructed volume, we propose to set *z*_0 _as the mean of the *Z *components of the new 3D landmarks

(30)

where *V*_0 _is the set of landmarks seen at image 0, *N*_0 _is the total number of these landmarks, and (**x**)_*z *_denotes the *Z *component of vector **x**. Finally, the aligned tilt series images is given by

(31)
